# Supplementary material for: Heuristic algorithms in evolutionary computation and modular organization of biological macromolecules: Applications to in vitro evolution
Source: PLoS One. 2022 Jan 27;17(1):e0260497. doi: 10.1371/journal.pone.0260497 (PMC8794168; doi:10.1371/journal.pone.0260497)
Supplement: S1 Fig — (PDF) [file pone.0260497.s001.pdf]

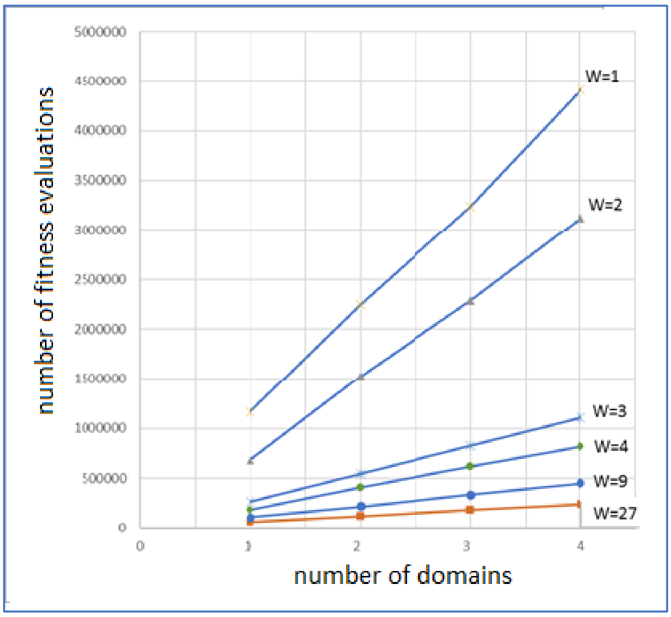

S1 Figure. Simple consensus-based BioRS: Series of RMHC tests with the BioRS function at varying W values. At W=27 the search is accelerated ~20 times. The starting position of the aptamer of interest is assumed to be located anywhere within the window of size *W*. We perform tests with different values of *W*. The search speed increases with increasing *W* as shown in Fig. S1 and S2. The search time is nonlinear, proportional to 1/W as illustrated in Fig. S2. Specifically, for the selected parameter values, when the window increases from a single-valued position to 220, the search speed increases by more than an order of magnitude.
