# Supplementary material for: Heuristic algorithms in evolutionary computation and modular organization of biological macromolecules: Applications to in vitro evolution
Source: PLoS One. 2022 Jan 27;17(1):e0260497. doi: 10.1371/journal.pone.0260497 (PMC8794168; doi:10.1371/journal.pone.0260497)
Supplement: S2 Fig — (PDF) [file pone.0260497.s002.pdf]

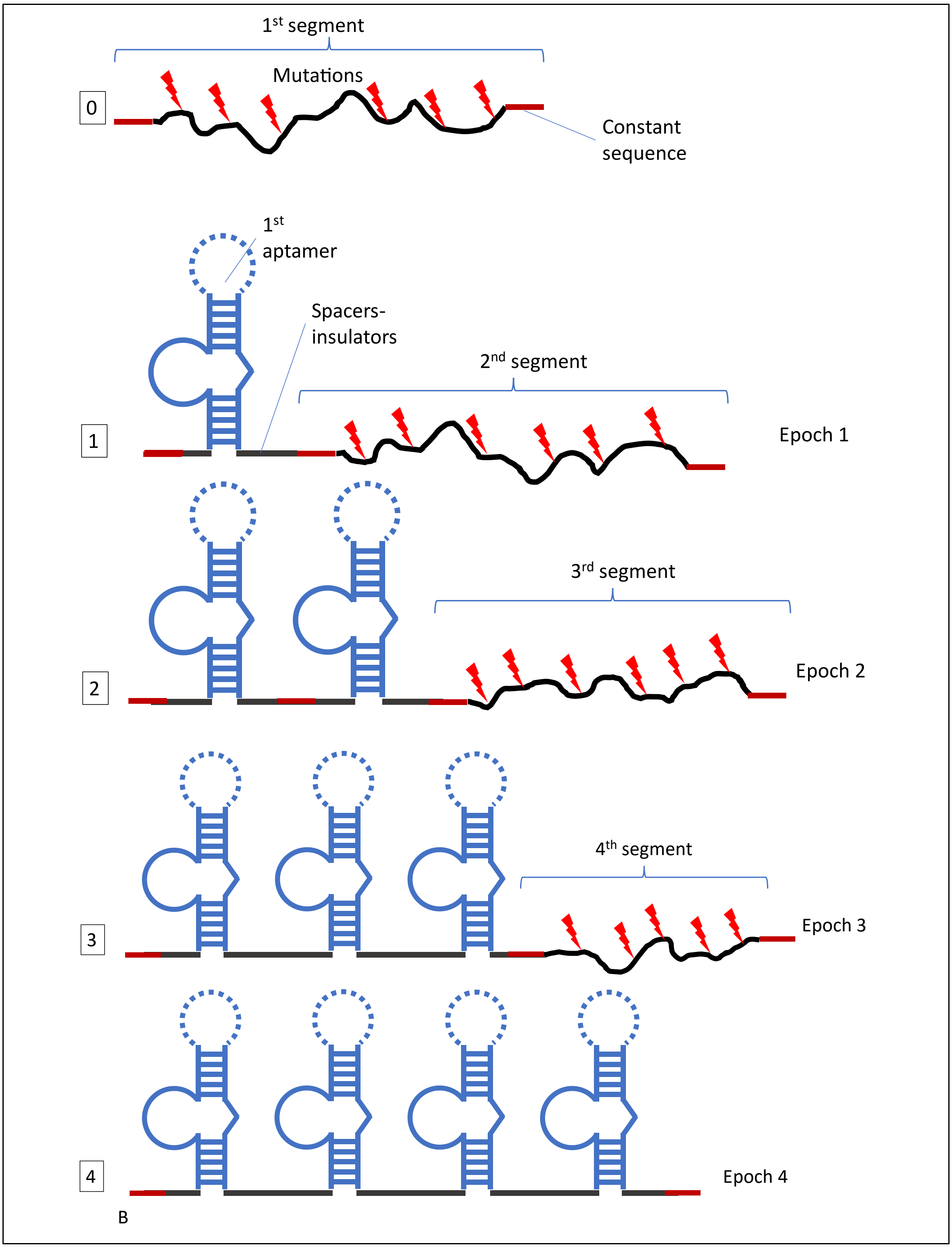

S2 Figure. Scheme of a local mutagenesis acting only on the last segment where the search for a new functional domain is performed at the current epoch. Local point mutagenesis is sketched by red lightning bolts. The rest of the sequence is not subjected to mutations.
