# Supplementary material for: Heuristic algorithms in evolutionary computation and modular organization of biological macromolecules: Applications to in vitro evolution
Source: PLoS One. 2022 Jan 27;17(1):e0260497. doi: 10.1371/journal.pone.0260497 (PMC8794168; doi:10.1371/journal.pone.0260497)
Supplement: S3 Fig — (PDF) [file pone.0260497.s003.pdf]

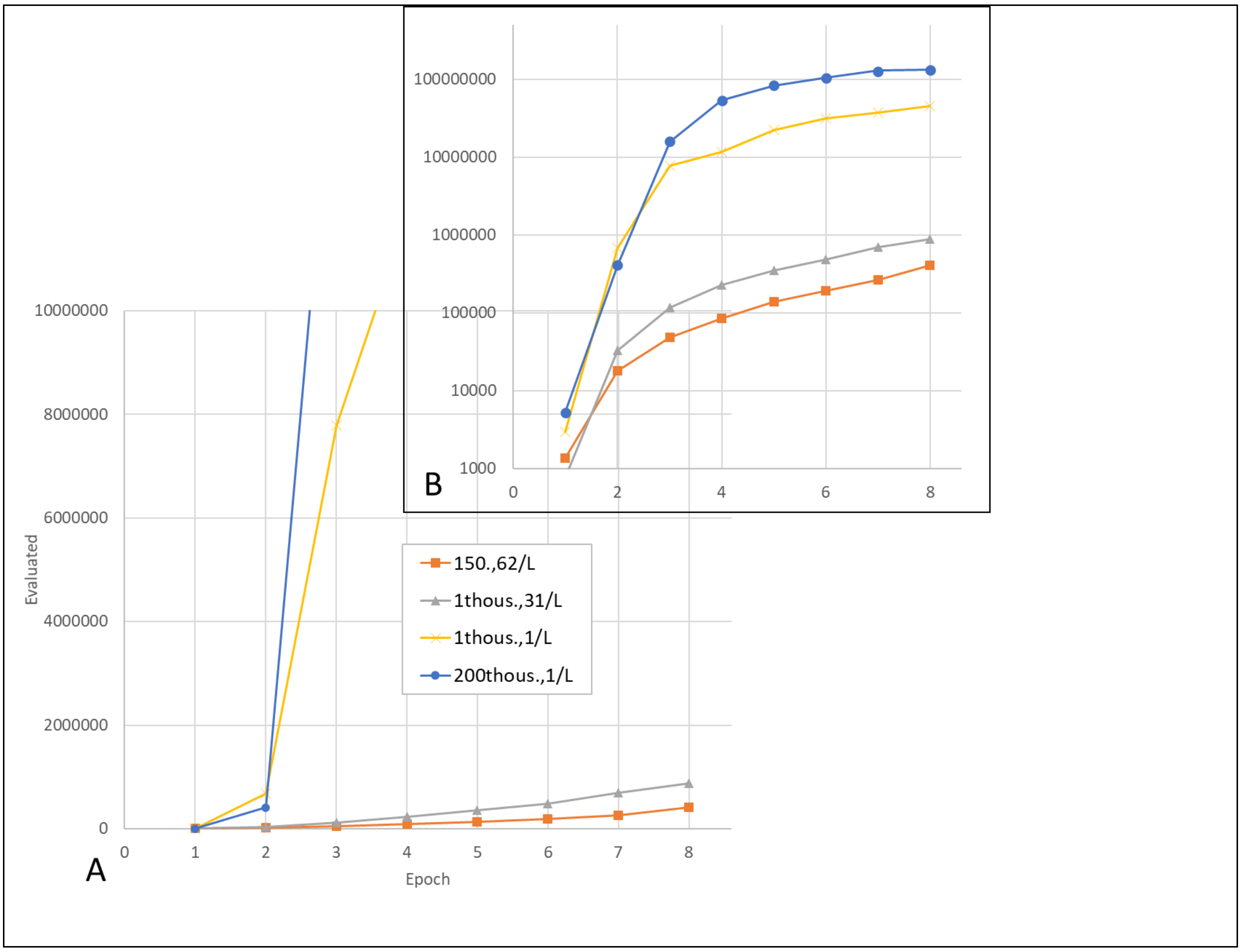

S3 Figure. For the BioRS GA the averaged time (in the average, the number of candidate string evaluations) to achieve the (n+1)th fitness level rises exponentially (A); plotting in semi-log scale confirms this observation (B)) (Cf [Nimwegen, Crutchfield, 2000; Nimwegen, Crutchfield, 2001]). For the first domain search it is not true because its finding in the initial population is highly probable that accelerates the search. Graphs are given for the runs from Table 1. Standard deviations are of value comparable to the mean. In the legend the curves are marked by the population size and mutation rate.

1. E. van Nimwegen, and J. P. Crutchfield, “Optimizing Epochal Evolutionary Search Population-Size Independent Theory,” Computer Methods in Applied Mechanics and Engineering, vol. 186, (No 2-4), pp. 171-194, 2000.
2. van Nimwegen E., Crutchfield J.P. Optimizing epochal evolutionary search population-size dependent theory, Machine Learning Journal. 2001. V. 45. P. 77-114.
