# Supplementary material for: Heuristic algorithms in evolutionary computation and modular organization of biological macromolecules: Applications to in vitro evolution
Source: PLoS One. 2022 Jan 27;17(1):e0260497. doi: 10.1371/journal.pone.0260497 (PMC8794168; doi:10.1371/journal.pone.0260497)
Supplement: S1 Table — (PDF) [file pone.0260497.s004.pdf]

**Parameters**	**# evaluations<br>(st. deviation)**	**Success rate<br>(%%)**
P = 1200; μ/λ = 0.4;<br>Nmut = 60	300 840.8 (199 733.08)	25
P =-3600; μ/λ = 0.4;<br>Nmut=40	759 248.16 (491 531.11)	35
P = 6000; μ/λ = 0.5;<br>Nmut = 18	1 343 557.22 (909 213.44)	45

S1 Table. GA effectiveness for competitive BioRS with (μ, λ) selection. (4 domains, 4 + 10 defined positions out of 26, W=220). Parameter values of μ/λ, mutation rates (Nmut is the mean number of mutations per chromosome), and population size, P, are chosen as the best in our preliminarily tests. Averaged over 40 runs.
