# Supplementary material for: Heuristic algorithms in evolutionary computation and modular organization of biological macromolecules: Applications to in vitro evolution
Source: PLoS One. 2022 Jan 27;17(1):e0260497. doi: 10.1371/journal.pone.0260497 (PMC8794168; doi:10.1371/journal.pone.0260497)
Supplement: S1 File — (PDF) [file pone.0260497.s005.pdf]

SUPPORTING INFORMATION

Heuristic algorithms in Evolutionary Computations and modular organization of biological macromolecules: applications to in vitro evolution

Alexander V Spirov, Ekaterina M Myasnikova

Royal Road functions

Introduction to RR & RS: Along with many other benchmark tests in EC, the RR fitness functions were invented to model the preservation and destruction of BBs by crossover operators imitating natural and test-tube evolution [van Nimwegen, Crutchfield, 2000; van Nimwegen, Crutchfield 2001]. These functions were devised so that to award fitness for the preservation of BBs. Four RR functions, of different complexity, were invented and introduced by Forrest, Mitchell, and Holland to specifically test crossover operations in GA [Forrest, Mitchell, 1993a;b; Mitchell et al., 1993]. The related Royal Staircase (RS) functions were devised and introduced by [van Nimwegen, Crutchfield, 2000].

In searching for fitness functions that are easy for GA and difficult for non-evolutionary methods, a whole family of increasingly complex RR functions was devised (R1, R2, R3, R4; [Forrest, Mitchell, 1993a; Mitchell, 1998]).

Though all the RR and RS theory is formulated in terms of binary strings, for the purposes of this publication, we will state the basics of the RR functions for the case of a four-letter alphabet.

The simplest among these functions, R1, is defined as a sequence of BBs, located end-to-end. The finding of each of blocks increases the problem fitness score by an additive value Δ. The BBs can be found in an arbitrary order. A BB is defined as a given sequence (word) of length K from the four-letter biological alphabet (A, T, G, & C). The value of fitness function R1 at already found K blocks is equal to by nΔ:

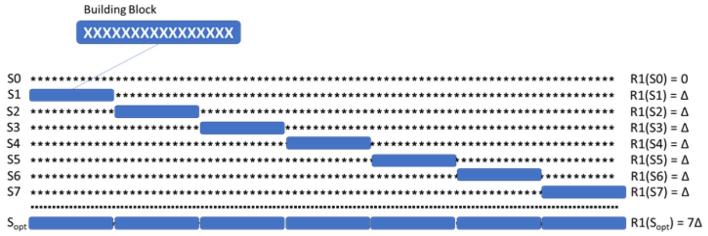

The maximum fitness value for this particular example is 7Δ.

R3 allows for random-letter spacers of the given length between BBs which do not contribute to the fitness score:

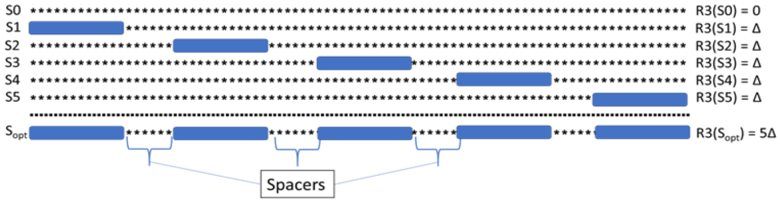

Royal Staircase functions: The RS class of fitness functions is a generalization of the RR

functions in which the subbasin-portal architecture is expressed in a more explicit form [van Nimwegen, Crutchfield, 2000]. The RS1 function is similar to R1, the main difference is that the order of the found BBs matters, that is, the line is built from left to right:

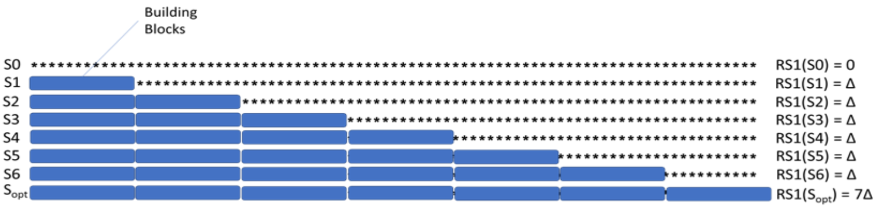

Finally, by analogy with the R3 function, we introduce the RS3 function, which has spacers between the BBs:

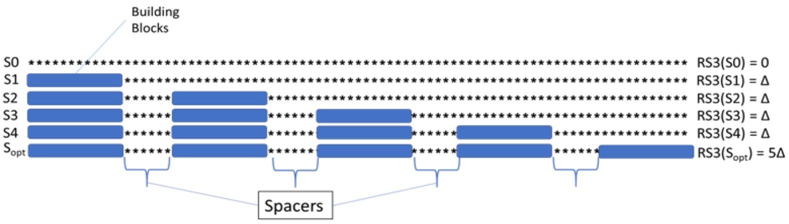

Because of the general parallels between RR & RS and the structure of biological macromolecules (RNA, DNA & polypeptides), we expect that our computational analysis of these functions will shed some light on the evolution of macromolecules and be useful as a theory for the forced molecular evolution.

References

1. E. van Nimwegen, and J. P. Crutchfield, "Optimizing Epochal Evolutionary Search Population-Size Independent Theory," Computer Methods in Applied Mechanics and Engineering, vol. 186, (No 2-4), pp. 171-194, 2000.
2. van Nimwegen E., Crutchfield J.P. Optimizing epochal evolutionary search population-size dependent theory, Machine Learning Journal. 2001. V. 45. P. 77-114.
3. Forrest S. & Mitchell M. (1993a). Relative building-block fitness and the building block hypothesis, In D. Whitley (ed.), Foundations of Genetic Algorithms, Vol.2, pp. 109-126, San Mateo, CA Morgan Kaufmann.
4. S. Forrest, and M. Mitchell (1993b), "Relative building-block fitness and the building block hypothesis," In D. Whitley (ed.), Foundations of Genetic Algorithms, vol. 2, pp. 109-126, San Mateo, CA Morgan Kaufmann.
5. Melanie Mitchell, John H. Holland, Stephanie Forrest: When will a Genetic Algorithm Outperform Hill Climbing. NIPS 1993: 51-58.
6. Mitchell, Melanie (1998). An Introduction to Genetic Algorithms. Cambridge, Massachusetts: MIT Press.
