# Supplementary material for: Heuristic algorithms in evolutionary computation and modular organization of biological macromolecules: Applications to in vitro evolution
Source: PLoS One. 2022 Jan 27;17(1):e0260497. doi: 10.1371/journal.pone.0260497 (PMC8794168; doi:10.1371/journal.pone.0260497)
Supplement: S2 File — (PDF) [file pone.0260497.s006.pdf]

Effectiveness of the evolutionary search for problems with variable domain positions

The noted above difference in the type of genotype-to-phenotype mapping in typical GAs and *in vitro* evolution makes it possible to significantly increase the search efficiency (at least in computational tests). The main idea here is that the evolutionary search should be carried out on sequences several times longer than the expected compact, or even the minimum, size of the required functional domain. Typically, in SELEX experiments, this is exactly the situation, where the random part of the molecule sequences in the initial pool significantly exceeds the functional domain found as a result [Bartel, Szostak, 1993; Lorsch, Szostak, 1994; Hager, Szostak, 1997; Ekland, Bartel, 1995].

We conducted tests for the selection scheme (μ, λ) (λ = 2400; μ / λ = 0.4 and mutation rate Pmut = 0.246 per bit per generation. Expectedly, with an increase in W, the speed of solving the evolutionary problem grows very quickly, especially rapidly at the beginning of an increase in W (inversely proportional to W). The results are shown in Figure below.

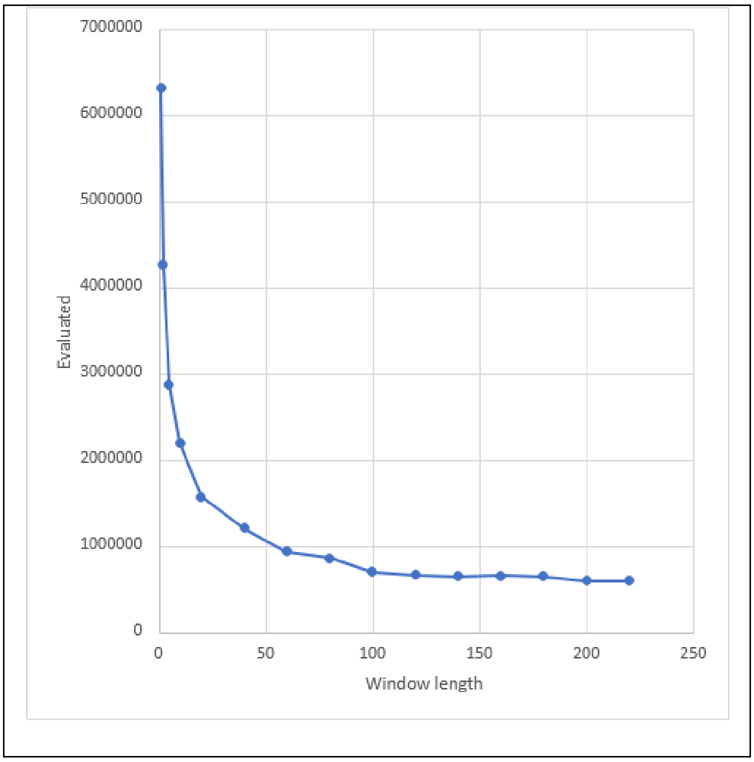

Figure. Search speed and efficiency growth versus W for our problem of evolutionary search with the simple consensus-based BioRS function. (μ, λ) selection (4 domains, 6+4 defined positions out of 26) at constant bitwise mutation rate.

References

1. Bartel DP, Szostak JW. Isolation of new ribozymes from a large pool of random sequences. Science. 1993 Sep 10;261(5127):1411-8.

2. Lorsch JR, Szostak JW., In vitro evolution of new ribozymes with polynucleotide kinase activity. Nature 1994; 371:31-36.

3. Hager AJ, Szostak JW., Isolation of novel ribozymes that ligate AMP-activated RNA substrates. Chem. Biol. 1997; 4:607-617.

4. Ekland EH, Bartel DP. The secondary structure and sequence optimization of an RNA ligase ribozyme. Nucleic Acids Res. 1995 Aug 25;23(16):3231-8.
