# Supplementary material for: Heuristic algorithms in evolutionary computation and modular organization of biological macromolecules: Applications to in vitro evolution
Source: PLoS One. 2022 Jan 27;17(1):e0260497. doi: 10.1371/journal.pone.0260497 (PMC8794168; doi:10.1371/journal.pone.0260497)
Supplement: Attachment — Submitted filename: ___Response_to_Reviewers.docx [file pone.0260497.s007.docx]

Dear Dr Bongard,

Thank you for the letter, regarding manuscript, ‘Heuristic algorithms in Evolutionary Computations and modular organization of biological macromolecules: applications to in vitro evolution ' by Spirov & Myasnikova. We hereby resubmit the manuscript.

Above all we would like to extend our thanks and appreciation to the editors and reviewer for making very important and helpful comments.

We modified the manuscript according to the reviewer’s recommendations. The major corrections are highlighted in red. Our point-by-point response to the reviewer’s comments follows. For the sake of convenience, the comments are enumerated. The page numbers are referred to according to the revised manuscript.

The manuscript was strongly revised, structured, more illustrations (two new figures), and references were added. Several figures were revised. In response to the Reviewer’s comments, Introduction and Discussion were restructured, Results section was shortened. The conclusions from our numerical tests were explicitly formulated.

The goal and major tasks of our study are now explicitly formulated in Introduction.

The most significant insertions have been introduced into Discussion. The reviewer drew our attention to the fact that the prospects for our research were not sufficiently elucidated`. In response to this comment, we have added extended paragraphs discussing the prospects for experimental implementation of the algorithms we have proposed and tested.

We hope to have given the full responses to all the comments.

Thank you for your consideration,

Sincerely,

Alexander Spirov

Ekaterina Myasnikova

PLOS ONE

Journal Requirements:

When submitting your revision, we need you to address these additional requirements.

1. Please ensure that your manuscript meets PLOS ONE's style requirements, including those for file naming.

Done.

2. Thank you for stating the following financial disclosure: 
 [The funders had no role in study design, data collection and analysis, decision to publish, or preparation of the manuscript].

At this time, please address the following queries:

a) Please clarify the sources of funding (financial or material support) for your study. List the grants or organizations that supported your study, including funding received from your institution.

The research was supported by the Russian Science Foundation (grant 17-18-01536).

b) State what role the funders took in the study. If the funders had no role in your study, please state: “The funders had no role in study design, data collection and analysis, decision to publish, or preparation of the manuscript.”

The funders had no role in study design, data collection and analysis, decision to publish, or preparation of the manuscript.

c) If any authors received a salary from any of your funders, please state which authors and which funders.
d) If you did not receive any funding for this study, please state: “The authors received no specific funding for this work.”

Please include your amended statements within your cover letter; we will change the online submission form on your behalf.

3. Thank you for stating the following in the Acknowledgments Section of your manuscript: 
[This work is supported by the Russian Foundation for Basic Research (grant no. 20-04-01015) and Federal Agency for Scientific Organizations of the Russian Federation (grant no. 01201351572).]
We note that you have provided funding information that is not currently declared in your Funding Statement. However, funding information should not appear in the Acknowledgments section or other areas of your manuscript. We will only publish funding information present in the Funding Statement section of the online submission form. 
Please remove any funding-related text from the manuscript and let us know how you would like to update your Funding Statement. Currently, your Funding Statement reads as follows: 
 [The funders had no role in study design, data collection and analysis, decision to publish, or preparation of the manuscript.]

Done.

Please include your amended statements within your cover letter; we will change the online submission form on your behalf.

Done.

4. Please amend either the abstract on the online submission form (via Edit Submission) or the abstract in the manuscript so that they are identical.

Done.


Reviewers' comments:

Reviewer #1:

1. It is difficult to determine if the work is technically sound as the authors need
to explain more clearly and specically what their motivations and goals were
for this research, or what questions they were looking to answer.

Our motivation: we, like some other researchers, are inspired by the similarity of ideas in the field of evolutionary computation and in vitro evolution, in general, and SELEX, in particular. We emphasis this in Introduction (*1.1 Biological in vitro evolution and genetic algorithms*; *1.2 Building Blocks in biology and genetic algorithms*) and Discussion. Moreover, we are inspired by our observation that well-known in GA foundations Royal Roads problem is very similar to the problem of evolutionary search from scratch for multidomain (and multifunctional) synthetic macromolecules. This remarkable similarity inspired us for this study. In Discussion we also discuss the ways and prospects of implementation of more effective algorithms into biological experiment that can be also regarded as our motivation.

In order to convey our motives to potential readers, we have added a new first figure (Fig. 1) to Introduction. This figure illustrates our expectation that "Heuristic algorithms from evolutionary computation hold remarkable promise for transfer to synthetic biology and biotechnology."

We have clearly formulated the goal and two major tasks of the article (last para in Introduction, p. 7).

2. How their methods were implemented in silico was not explained clearly, and they do
not summarize what specific conclusions they have drawn from their results.

We expanded the description of the methods and their specific implementation in the Methods section. Specifically, we added the details of our implementation of mutations and crossover.

The detailed description of standard selection methods ((µ, λ) and Sigma scaling selection) and specific versions of BioRS (biologically substantiated versions of BioRS fitness function) was previously included into Methods.

To bring the BioRS function notations into correspondence with the rest of the text, the last subsection is now entitled “Competitive BioRS function (with domain interference)” and the explanation: “This version of the BioRS function is a further extension of “BioRS function with alternative domain structures” in which the domain interference is additionally taken into account” is introduced.

To make the application of these methods clearer for readers, we have introduced several relevant links to this section from those places where the corresponding method is used.

We have formulated the main conclusions summarizing the results of our numerical tests in the end of the Results section (pp. 28-29). We have also significantly expanded and restructured the Discussion section where these conclusions are analyzed and discussed (see also our responses below).

3. For example the authors reference the SELEX method, an existing in vitro
method for evolution DNA aptamers. What are they specifically hoping to
contribute to methods such as SELEX?

We have added extended paragraphs to Discussion, where we formulate the prospects of experimental implementation of the algorithms we have tested. Specifically, we discuss modern experimental approaches of in vitro evolution using nucleic acid synthesizers, as it was done by prof. Kell’s group [Knight et al., 2009] (3.3 “In in vitro evolution small populations are preferable”, final paragraph (p.31)), and microfluidic techniques (3.4 “Prospects of hill-climbing-based methods” final paragraphs (pp.32-33)).

4. In the methods section they summarize various evolutionary algorithms,
but they do not explain how the authors themselves explicitly applied them
in their experiments. It is also not clear from the methods section how these
various algorithms which they discuss related to their research questions or
goals.

The Methods section only encompasses those algorithms that are tested in our study. Now we specify these algorithms explicitly: “Our tests were implemented for two versions of BioRS: simple consensus-based and competitive.” To avoid the confusion the notation of the BioRS function with domain interference is corrected to “Competitive BioRS with domain interference”. See also the response to the previous comment.

Our initial selection of (new) heuristic algorithms for in silico testing was inspired and dictated by the RR research area in GA. It was there that the standard (mu, lambda)-GA was tested in comparison with Sigma scaling selection, and in comparison with RMHC. We emphasize this in the text (See, for example, 2.2, 2^nd^ last para, p.22). To develop this issue, we have also proposed and tested a parallel RMHC. A more complete list of promising algorithms that we have not tested yet, but which have shown their effectiveness in GA for RR problems, is given in Discussion. We expect that algorithms that are effective for RR problems in GA may turn out to be promising for in vitro evolution problems (which we repeatedly emphasize in the text). We have restructured the Results section to make it clearer for the reader. We have also paid attention to this issue in Discussion (final subsection).

5. The authors should define clearly in their introduction what questions
they are seeking to answer with this research, what their novel contributions
were, and what their utility is.

The Introduction section has been substantially modified and structured to clearly outline our motives, our goal and the tasks we solve, as well as our most important result. As the most significant new result, we interpret our conclusion about the prospects of implementing the RMHC & parallel RMHC algorithms in new experimental approaches.

6. The results section is very verbose and contains a signiant discussion
of related work, so it is difficult to determine what questions the various
experiments or case studies were seeking to answer and whether they did so
successfully. The authors do not clearly dene what success means in their
various experiments, and they need to explain more clearly what the exact
tness function was, how it was evaluated, and how it was implemented in
silico. It is therefore difficult to identify what conclusions can be drawn from
the paper.

We have somewhat shortened or completely removed to Discussion the introductory paragraphs in Results subsections to present our work and its results more clearly. We explicitly presented our fitness function in Methods and explained how it was evaluated, and how it was implemented in silico. We have added key conclusions at the end of the Results (pp. 28-29) and discussed them in detail in the new Discussion subsections (see below).

7. It was only evident after reading the entire results section that the point
of the in silico experiments was to identify the optimal algorithm features
and hyperparameters for in vitro implementation through in silico simulation.
This should be made clear in the introduction.

Our intentions, goals and objectives are now clearly and explicitly formulated in the end of Introduction, p.7 (as noted above).

8. The discussion also contains a large discussion of related work rather
than a discussion of their experimental results, and so after reading it, it is
still unclear what conclusions the authors have drawn from the research, or
whether their conclusions are novel, and what their utility is.

The authors need a separate section where they summarize their conclusions,
to discuss how their results lead to those conclusions, and connect their
conclusions back to the goals or questions which they were hoping to answer
with this research.

As already mentioned above, we substantially supplemented and structured Discussion to discuss the results of our numerical tests and conclusions from them. The novelty of our research is determined by the novel idea of introducing the biologically substantiated extensions of RS-type fitness function that have prospects in experimental applications. This is explained in Introduction.

9. The experiments conducted in the paper were all in silico simulations. These
simulations were composed of an analysis of their proposed evolutionary
algorithm from different perspectives. Therefore, the expected statistical analysis
would involve typical metrics often reported when analysing the performance
of evolutionary algorithms.

When analyzing the performance of evolutionary algorithms one generally
needs to report:

-Some variation of average success rate, that is, the fraction of algorithm
runs where a solution of satisfactory quality was obtained.

-The mean best fitness, which is the optimal fitness obtained from each
run averaged over the number of runs.

-The effectiveness, or the average solution quality obtainable within a
given computational limit.

-Or the efficiency, which is the average amount of time needed to achieve
a solution of satisfactory quality. Given that computational time depends
on the available hardware, efficiency is often reported as the
average number of evaluations to a solution.

These are also usually accompanied by progress curves for the various
metrics. The authors reported success rate, effectiveness and efficiency in
various figures throughout the results section. These reported metrics are
sufficient for the nature of their in silico experiments, however, it is not
always made clear how success was defined, that is, how the authors defined
a solution to be of acceptable quality.

We have completed all the Tables, as well as the corresponding figures (Fig. 7; 8; 9) so that all the test criteria adopted in the GA are given explicitly. Specifically, when comparing algorithms, we give the average number of fitness evaluations, standard deviation, and success rate.

Additionally, we present in Supplement and briefly analyze the graphs presenting the mean duration (# of evaluations) of each next epoch in GA tests at different parameter values. These tests (Table 1) were implemented for the specific BioRS version with eight domains to make this empirical analysis more illustrative.

10. There are many grammatical problems with the text, potentially because the
author's rst language is not English. So much so that it makes it difficult
to understand what the authors are trying to communicate in many places.

The spell check is done, grammar is corrected.

11. Further, some concepts were not sufficiently claried. The following concepts
need to be explained more clearly:

-Directed evolution / artificial evolution of biological macromolecules.

The definition is given by an endnote.

-How evolution of biological macromolecules is directed in vitro.

This same endnote also provides a response to this comment.

-In the case studies, how were the molecules which were being evolved,
represented and evaluated in silico.

As we explained now in the Methods section, in our tests: potential solutions are represented by one-dimensional symbol strings in (A,T,G,C) four-letter alphabet; bitwise mutations, (µ, λ) selection, and single point crossover are applied. Fitness functions for simple consensus-based and competitive BioRS are defined below in this section (p.36).

-Implementation details of the selection, mutation and crossover /
recombination methods.

Implementation details are now given in Methods (pp.36-37).

12. Regarding the layout of the paper, the research questions or goals of the
research need to be clearly stated in the introduction. The introduction
should also clearly describe what this research contributes to the field.

Done. Please see our responses above.

13. Discussions of related work should be separated from the results and discussion
sections and moved to a separate related work section. The results section
should clearly explain what experiments were run and what results were ob-
tained from each experiment. The discussion section should discuss these
results, not related work, and extract conclusions from them. The conclu-
sions section should summarize everything which can be extracted from the
results and connect the conclusions back to the goals and questions of the
research.

As discussed above, the main sections of the article, in particular Discussion, are modified, restructured in response to the Reviewer’s comments.

6. PLOS authors have the option to publish the peer review history of their article ([what does this mean?](https://journals.plos.org/plosone/s/editorial-and-peer-review-process#loc-peer-review-history)). If published, this will include your full peer review and any attached files.


If you choose “no”, your identity will remain anonymous but your review may still be made public.
==================================================================
